# A Case of Sarcoid Uveitis Diagnosed With Mammography Two Months After Normal Chest Imaging

**DOI:** 10.1155/crop/8871004

**Published:** 2025-02-12

**Authors:** Madison Riccardi, Robert Contento, Cory Christensen, Amy Brady, Rebecca L. Swan, Robert T. Swan

**Affiliations:** ^1^Norton College of Medicine, SUNY Upstate Medical University, Syracuse, New York, USA; ^2^Department of Ophthalmology & Visual Sciences, SUNY Upstate Medical University, Syracuse, New York, USA; ^3^Department of Pathology, SUNY Upstate Medical University, Syracuse, New York, USA; ^4^Department of Radiology, SUNY Upstate Medical University, Syracuse, New York, USA

**Keywords:** mammography, sarcoidosis, uveitis

## Abstract

**Purpose:** Sarcoidosis is a systemic inflammatory disease associated with ocular involvement in 20%–30% of cases. The current gold standard for detecting sarcoidosis is computed tomography of the thorax, which is 73% sensitive. Definitive diagnosis necessitates biopsy, with Schaumann bodies and non-necrotizing granulomas serving as key pathological hallmarks.

**Observations:** Our patient, a 44-year-old White female, presented for a second opinion on her bilateral chronic intermediate uveitis with intractable chronic cystoid macular edema of the left eye. Our clinical suspicion for sarcoidosis was high, but the computed tomography thorax scan did not show any abnormal findings. A routine mammogram completed 4 weeks prior to our initial evaluation showed axillary lymph node enlargement with calcifications. Subsequent biopsy was consistent with sarcoidosis. Treatment with mycophenolate mofetil resolved the uveitis and macular edema.

**Conclusions and Importance:** The diagnosis of sarcoidosis can be challenging due to nonspecific ocular signs and the potential for falsely negative findings on imaging. This case highlights the importance of patient education and self-surveillance regarding the characteristic systemic symptoms of sarcoidosis, which commonly involve the lungs, eyes, skin, joints, etc. Our report demonstrates the significance of maintaining a high level of suspicion for sarcoidosis in patients with characteristic ocular findings, even when initial imaging results are negative or inconclusive.

## 1. Introduction

Sarcoidosis is a systemic inflammatory disease characterized by the presence of noncaseating epithelioid giant cell granulomas, most commonly in the hilar and mediastinal lymph nodes. The disease also frequently involves the eyes, skin, heart, and nervous system. Ocular involvement occurs in 20%–30% of cases, mainly affecting the uveal tract (sarcoid uveitis). Ocular involvement in patients with sarcoidosis develops more commonly in females and in Black patients. The age distribution of ocular sarcoidosis is bimodal, with peaks at 20–30 and 50–60 years [1].

Given the diverse manifestation of sarcoidosis, clinical suspicion often plays an important role in making an accurate diagnosis. Non-ophthalmic characteristic findings include the presence of pulmonary symptoms and elevated angiotensin-converting enzyme (ACE) [2, 3]. In cases of high clinical suspicion, a computed tomography (CT) of the thorax is a useful diagnostic tool [4]. Definitive diagnosis of sarcoidosis requires compatible clinical presentation, evidence of noncaseating granulomatous inflammation seen on biopsy, and the exclusion of other diseases with similar features [2]. We report a case of sarcoid uveitis diagnosed after biopsy of an enlarged axillary lymph node identified through calcifications on mammogram, despite a negative contemporaneous CT thorax, underscoring the importance of staying vigilant in patients with a high pretest probability of sarcoidosis.

## 2. Case Presentation

A 44-year-old White female was referred to our clinic for a second opinion on chronic cystoid macular edema (CME) of the left eye (OS). Her ocular history was significant for a 10-year history of chronic intermediate uveitis of both eyes (OU) treated with chronic prednisolone drops. The macular edema on initial presentation had improved with a sub-Tenon triamcinolone injection. The CME had recurred 18 months prior to referral and had not improved with a series of bevacizumab intravitreal injections.

**Figure 1:**
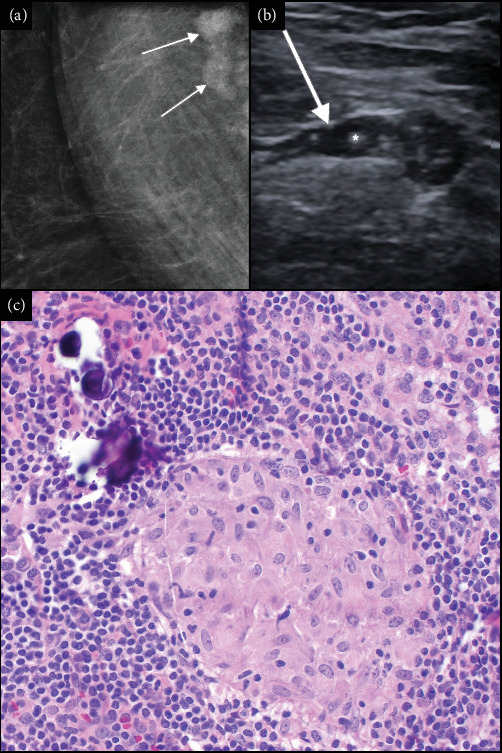
(a) Diagnostic mammogram of the axillary tail of the right breast showing axillary lymph nodes with internal microcalcifications. (b) Ultrasound of the axillary lymph node (arrow) demonstrating calcifications within the cortex (asterisk). (c) (400× magnification) A representative image from one of the lymph nodes found within the axillary excision specimen that shows a well-formed, non-necrotizing granuloma and a Schaumann body, that is, a giant cell with ingested concentric calcifications, seen at the top left.

While the patient had no relevant past medical history at our initial ophthalmic evaluation, she did note that she was in the process of being worked up for possible breast cancer. Eight weeks prior to that initial evaluation, a routine screening mammogram had suggested prominent lymph nodes with some calcifications on the right axilla, findings that were absent in both screening mammograms from the previous 4 years. These findings were then confirmed 2 weeks later on a follow-up diagnostic mammogram (Figure 1(a)) and ultrasound (Figure 1(b)). Five weeks later, a CT thorax was performed and did not show any abnormalities, including no evidence of axillary lymphadenopathy or pulmonary nodules.

**Figure 2:**
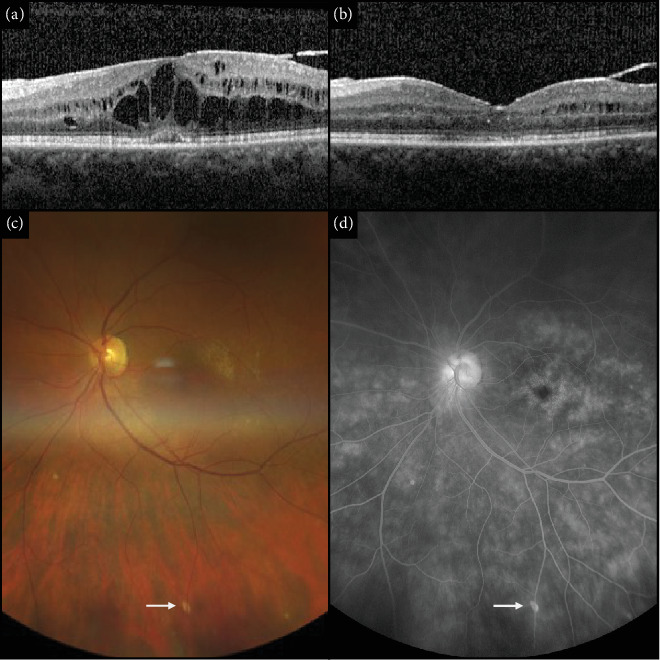
(a) Optical coherence tomography (OCT) of the left macula showing cystoid macular edema (CME) prior to initiating mycophenolate mofetil treatment. (b) OCT macula of the left eye showing improvement of CME 2.5 months after initiating mycophenolate mofetil treatment. (c) Fundus photograph showing the area of atrophy under the retinal vein (arrow) and (d) intravenous fluorescein angiogram of the left eye before initiating mycophenolate mofetil treatment.

Our initial ophthalmic exam revealed no anterior chamber inflammation but trace vitreous haze and 1+ vitreous cell OU, according to the Standardization of Uveitis Nomenclature criteria [5]. The right eye (OD) had a mild nuclear sclerotic cataract, and OS was pseudophakic. The peripheral exam was significant for small areas of punctate atrophy underneath the venous arcades (Figure 2(c)). Optical coherence tomography (OCT) of the macula demonstrated CME OU, greater OS (Figure 2(a)). Fluorescein angiography confirmed CME OU and revealed optic nerve leakage with diffuse, patchy leakage in the periphery of OU. No vasculitis was observed (Figure 2(d)).

She did not have tattoos nor any notable skin, pulmonary, or systemic symptoms on review of systems. Laboratory testing was positive for human leukocyte antigen (HLA)-B8 and an ACE level of 67 (reference range 12–68). Negative results were obtained for fluorescent treponemal antibody absorption, Lyme antibodies, and interferon-gamma release assay.

At that time, the patient was informed that while her uveitis was considered idiopathic, we strongly suspected sarcoidosis and told her to alert us of any changes in her health. Methotrexate was considered but was contraindicated due to the patient's alcohol consumption. We recommended she consider mycophenolate mofetil for her chronic uveitis. She declined, preferring to wait for the results of the breast cancer workup first.

Seven weeks after our initial exam, a biopsy of the right axillary lymph node revealed multiple small non-necrotizing granulomas and hyalinized fibrous tissue with calcifications (Schaumann bodies), consistent with sarcoidosis (Figure 1(c)). The patient returned for ophthalmic follow-up 6 weeks later and reported the biopsy results. She recalled being told by the breast center that “it was not cancer,” but no subsequent follow-up had been arranged. The lymph node pathology report was reviewed and, with her associated eye findings, she was diagnosed with sarcoidosis. At that time, she was referred to the sarcoidosis clinic where additional workup including repeat CT thorax and chest X-ray (CXR), each remaining normal. No other systemic evidence of sarcoidosis was found. EKG done at her primary care physician's office was normal.

The patient was subsequently started on mycophenolate mofetil 1 g orally twice daily. This controlled the uveitis and led to resolution of the CME over the following 2.5 months. The eye inflammation and CME remained controlled for the next 25 months, at which point she stopped the mycophenolate mofetil, and now continues to be stable 3 months later (Figure 2(b)). She has been followed at the sarcoidosis clinic every 6 months, and to date, no involvement in any other organ systems has developed.

## 3. Discussion

Ocular sarcoidosis is often difficult to diagnose due to nonspecific ocular inflammatory signs. There are many systemic findings suggestive of sarcoidosis. In addition to the typical pulmonary review of systems, we ask each patient about enlarged lymph nodes, skin rash, and periodic tattoo swelling. Our patient had chronic intermediate uveitis with peripheral atrophy under the retinal veins and workup negative for other etiologies. While she did have several characteristic intraocular signs (snowballs OU, inflammation OS > OD, and bilaterality), she did not quite meet the criteria for a probable diagnosis of ocular sarcoidosis. This requires an elevated ACE, according to revised criteria published by the International Workshop on Ocular Sarcoidosis [6]. Finally, our patient was positive for HLA-B8, which is associated with sarcoidosis in White patients [7].

Definitive diagnosis of sarcoidosis relies on a biopsy to demonstrate evidence of its pathological hallmark: well-formed non-necrotizing granulomas [2]. Crystalline inclusions, composed predominantly of calcium oxalate, are frequently found in the giant cells that comprise granulomas in sarcoidosis [8]. They serve as the nidus for calcium deposition leading to the formation of Schaumann bodies, which are large, concentric calcifications that contain calcium oxalate crystals [8].

Although our patient did not have any conjunctival lesions, a conjunctival biopsy is a simple procedure that can allow for tissue diagnosis of sarcoidosis [4]. In the absence of conjunctival lesions or characteristic skin rash, attention is usually shifted to the chest imaging to evaluate for hilar lymphadenopathy. While CXR is a low-cost intervention, studies have reported a higher false negative rate in detecting sarcoidosis among White women over 50 years of age [9, 10]. CT thorax provides better evaluation of lymphadenopathy and pulmonary involvement because it shows improved anatomical lung detail and is far more sensitive in detecting parenchymal, mediastinal, and hilar structures, allowing for biopsy planning [11]. Compared to CXR, CT thorax is found in many studies to have higher sensitivity (73% compared to 68%) in detecting bilateral hilar lymphadenopathy (BHL). BHL, either by CT thorax or CXR, is considered the most sensitive investigational finding for ocular sarcoidosis criteria [4].

Fluorodeoxyglucose-positron emission tomography/computed tomography (FDG-PET/CT), which indicates increased glucose uptake by macrophages and lymphocytes to demonstrate active sites of inflammation, is also useful in diagnosing sarcoidosis [4]. It has a sensitivity and specificity of 85.7% and 95.5%, respectively, and has been shown to enable the diagnosis of intraocular sarcoidosis even in patients with a normal CT scan [4].

Distinctive to this case, an abnormal contemporaneous mammogram began an unusual path that led to the discovery of sarcoidosis through a biopsy of the axillary lymph nodes. Sarcoidosis of the axillary lymph node is very rare, occurring in less than 1% of cases [12]. Breast involvement and axillary lymphadenopathy are often confused with benign or malignant tumors [13].

While generally useful in detecting sarcoidosis, CT thorax may still yield a false negative result, as granulomas can be located elsewhere than the lungs. Therefore, a high index of suspicion should be maintained when characteristic symptoms of sarcoid uveitis are established, even after negative CT thorax. Of uveitis patients who progress to systemic sarcoidosis, 50% do so within the first 5 years of uveitis onset [14]. Therefore, it is recommended that patients be monitored through clinical assessments, imaging studies such as CXR and CT scans, pulmonary function tests, bronchoscopy with biopsy when necessary, and laboratory tests such as ACE levels for at least 5 years after diagnosis [14].

This report expands the known diagnostic scope of sarcoid uveitis by utilizing additional modes of imaging, such as the mammogram, to identify granuloma deposition. We demonstrate that negative findings on CT thorax should not rule out the investigation of possible sarcoidosis. Had this patient not been prompted to report on unusual systemic symptoms, her diagnosis may have been delayed. In addition, we make the case that the physician should take care to periodically expand their interviews beyond the ocular history to also include a systemic inventory of their patients. While our findings will not apply to all cases, they emphasize the importance of vigilance in clinical practice. Although mammograms are not advocated as routine diagnostic tools for sarcoidosis, our report highlights the significance of investigating abnormal findings in patients with suspected systemic abnormalities.

Despite the inherent challenges in recognizing sarcoidosis, it is important to appreciate that there is no definitive method of exclusion in its diagnosis. While there are various approaches to detecting this pathology, negative test results or the absence of specific findings should not immediately rule out sarcoidosis from the differential diagnosis. Sarcoidosis requires close observation and evaluation when there is clinical suspicion, as prompt diagnosis and intervention are crucial for improved outcomes and management.

Clinicians should maintain a high index of suspicion for sarcoidosis in patients presenting with chronic uveitis, especially when accompanied by systemic signs such as peripheral punctate atrophy or unexplained systemic symptoms. In cases where CT thorax is noncontributory, patient education, self-surveillance, and periodic reviews of interval medical history may aid diagnosis.

## Data Availability

Data sharing is not applicable to this article as no new data were created or analyzed in this study.

## Nomenclature

SUNY: State University of New York
CT: computed tomography
ACE: angiotensin-converting enzyme
CXR: chest X-ray
CME: cystoid macular edema
OU: both eyes
OD: right eye
OS: left eye
HLA: human leukocyte antigen
FDG-PET/CT: fluorodeoxyglucose-positron emission tomography/computed tomography
OCT: optical coherence tomography
